# Toll-Like Receptor 2 Targeted Rectification of Impaired CD8^+^ T Cell Functions in Experimental *Leishmania donovani* Infection Reinstates Host Protection

**DOI:** 10.1371/journal.pone.0142800

**Published:** 2015-11-11

**Authors:** Syamdas Bandyopadhyay, Santanu Kar Mahapatra, Bidisha Paul Chowdhury, Mukesh Kumar Jha, Shibali Das, Kuntal Halder, Suchandra Bhattacharyya Majumdar, Bhaskar Saha, Subrata Majumdar

**Affiliations:** 1 The Division of Molecular Medicine, Bose Institute, Kolkata, India; 2 National Centre for Cell Science, Ganeshkhind, Pune, India; Jawaharlal Nehru University, INDIA

## Abstract

*Leishmania donovani*, a protozoan parasite, causes the disease visceral leishmanisis (VL), characterized by inappropriate CD8^+^ T-cell activation. Therefore, we examined whether the Toll-like Receptor 2 (TLR2) ligand Ara-LAM, a cell wall glycolipid from non-pathogenic *Mycobacterium smegmatis*, would restore CD8^+^ T-cell function during VL. We observed that by efficient upregulation of TLR2 signaling-mediated NF-κB translocation and MAPK signaling in CD8^+^ T-cells (CD25^+^CD28^+^IL-12R^+^IFN-γR^+^), Ara-LAM triggered signaling resulted in the activation of T-bet, which in turn, induced transcription favourable histone modification at the IFN-γ, perforin, granzyme-B promoter regions in CD8^+^ T-cells. Thus, we conclude that Ara-LAM induced efficient activation of effector CD8^+^ T-cells by upregulating the expression of IFN-γ, perforin and granzyme-B in an NF-κB and MAPK induced T-bet dependent manner in VL.

## Introduction

Visceral leishmaniasis caused by the protozoan parasite, *Leishmania donovani*, is fatal, if untreated. Dysfunctions of macrophages and T-cells during VL result in severe immunosuppression [1–3]. CD4^+^ T-cells activation is essential for IFN-γ-mediated protection against leishmaniais [4] whereas CD8^+^ T-cells confer protection via perforin, granzyme-B-mediated direct killing of *Leishmania*-infected host cells [5–6]. *L*. *donovani* induces functional exhaustion of CD8^+^ T-cells [7]. Activation of *Leishmania*-specific CD8^+^ T-cells is indispensable for clearance of the pathogen. For naive CD8^+^ T cells’ antigen-specific activation and differentiation, signals from T-cell receptor, CD28, IL-12-induced IFN-γ and TLR2 are reqiured [8–12]. The transcription factor T-bet promotes naive CD8^+^ T-cell differentiation to effector cytotoxic T lymphocytes (CTL) [13–14], expressing CD25 (IL-2R-α), IL-12 receptor (IL-12R) and IFN-γR.

TLR2, forming heterotypic associations with TLR1 or TLR6, recognizes triacetylated or diacetylated lipopeptides, respectively [15–17]. Ara-LAM, a cell wall glycolipid of *Mycobacterium smegmatis*, has been reported to confer protection against leishmanial pathogenesis via TLR2-dependent induction of the proinflammatory responses [18]. Ara-LAM–induced activation of p38 MAPK signalling in *Leishmania* infected macrophages shifts their Th2 phenotype towards Th1 via chromatin modification at various proinflammatory cytokine gene loci [19]. However, it was unclear if Ara-LAM would modulate TLR2 signalling in CD8^+^ T-cells, which might play a potential role in the regression of leishmanial pathogenesis.

In this study, we have demonstrated that Ara-LAM drives the activation of CD8^+^ T-cells, which are CD25^+^CD28^+^IL-12R^+^IFN-γR^+^ and specifically destroys the *L*. *donovani*-infected macrophages. Ara-LAM enhances T-bet expression in CD8^+^ T-cells via upregulation of NF-κB and p38MAPK in a TLR2-dependent pathway. T-bet ultimately enhances the expression of IFN-γ, perforin, and granzyme-B in CD8^+^ T-cells via histone modifications at their respective promoter regions to restore host-protective CD8^+^ T-cell responses.

## Materials and Methods

### Reagents and chemicals

RPMI-1640 medium, penicillin and streptomycin, SB203580 (p38MAP Kinase inhibitor), SN50 (NF-κB inhibitor) were from Sigma (St. Louis, MO, USA). dNTPs, Revert Aid M-MuLV Reverse Transcriptase, oligo dT, RNase inhibitor and others for cDNA synthesis were from Fermentas (Ontario, Canada). Phospho-H3 and acetyl-H3 Abs were from Abcam (Cambridge, UK) and chromatin immunoprecipitation (ChIP) assay kit was from Millipore (Bedford, MA). TLR2, MyD88, IRAK 1, NF-κB, p38, phospho-p38, ERK1/2, phospho-ERK1/2, T-bet, β-Actin antibodies were from Santa Cruz Biotechnology (Texas, USA). Ara-LAM was isolated as previously described [20]. Lipopolysaccharide contamination (<25 ng/mg) was checked by the Limulus test. All antibodies for FACS were from BD Biosciences (San Diego, USA).

### Animals and parasites

*L*. *donovani* strain AG-83 (MHOM/IN/1983/AG-83) was maintained *in vitro* in Medium-199 (Sigma, St. Louis, MO) with 10% FCS (Gibco-BRL) and virulence was maintained by passage through BALB/c mice. Stationary-phase promastigotes obtained by suitable transformation were used for experiments [21]. BALB/c mice were infected with stationary phase *L*. *donovani* promastigotes (i.v., 2×10^7^/mouse). BALB/c mice (6–8 weeks, NCLAS, Hydrabad, India) were divided into the following experimental groups: (1) control (receiving PBS); (2) infected (receiving *L*. *donovani*); (3) Infected and Ara-LAM–treated infected (Ara-LAM 30μg/kg body weight-injected 2 days prior to infection); (4) control shRNA and (5) TLR2 shRNA (TLR2 shRNA or control shRNA [bearing scrambled sequence] treatment 72h before Ara-LAM treatment). Mice were sacrificed on 14 and 28 days after infection by cervical dislocation method as mentioned by Institutional Animal Ethical Committee (Bose Institute), bearing a registration number: 1796/PO/ERe/S/14/CPCSEA. This study followed the Institutional Animal Ethical Committee approval. *L*. *donovani* infection was expressed in Leishman-Donovan units.

### Isolation and purification of macrophages and CD8^+^ T-cells

Thioglycolate-elicited (i.p., 4% w/v, 1.0 ml/mouse) macrophages from different experimental groups of BALB/c mice were infected with stationary phase *Leishmania* promastigotes at a ratio of 1:10 [22]. Splenic CD8^+^ T-cells (purity >99% as ascertained by FACS) from the indicated mice were isolated by positive selection using CD8^+^ IMag beads, according to the manufacturer’s instructions (BD Biosciences). CD8^+^ T-cells were cultured in RPMI-1640 with plate-bound anti-CD3ε (5μg/mL) and CD28 (1μg/mL).

### Preparation of TLR2 and T-bet-specific siRNA

TLR2 and T-bet-specific siRNA were synthesized using the Silencer siRNA Construction kit (Ambion). Scrambled siRNA was synthesized with the similar GC content. Silencing primers are listed in the Table 1.

**Table 1:** Sequences of the PCR primers.

Gene	Sequences
**TLR2**	FP 5’- TCTGGGCAGTCTTGAACATTT -3’
	RP 5’- AGAGTCAGGTGATGGATGTCG -3’
**IFN-γ**	FP 5’- AGCTCTTCCTCATGGCTGTTTC -3’
	RP 5’- TGTTGCTGATGGCCTGATTGT -3’
**Perforin**	FP 5’- CTGAGCGCCTTTTTGAAGTC -3’
	RP 5’- AAGGTAGCCAATTTTGCAGC -3’
**Granzyme-B**	FP 5’- CTCTCGAATAAGGAAGCCCC -3'
	RP 5’- CTGACCTTGTCTCTGGCCTC -3'
**T-bet**	FP 5’- CCTCTTCTATCCAACCAGTATC -3’
	RP 5’- CTCCGCTTCATAACTGTGT -3’
**IFN-γ promoter**	FP 5’- GAGAAATTCACATTACAAGGGC -3’
	RP 5’- TTAAGATGGTGACAGATAGGTGG -3’
**Perforin promoter**	FP 5’- GTACTAGCCTGCTCAAACCT -3’
	RP 5’- CTAATCACAGTGTCCCATGAG -3’
**Granzyme promoter**	FP 5’- ATGCTCCTGATTACCCTCAC -3’
	RP 5’- CAGAGAACCACCACTTACAG -3’
**TLR-2 siRNA**	FP 5’-AAAGAGAAAGTACTTACTGCACCTGTCTC-3’
	RP 5’- AATGCAGTAAGTACTTTCTCTCCTGTCTC -3’
**T-bet siRNA**	FP 5’- AAACAAACATCCTGTAATGGCCCTGTCTC -3’
	RP 5’-AAGCCATTACAGGATGTTTGTCCTGTCTC-3’
**GAPDH**	FP 5’- GTTGTCTCCTGCGACTTCAACA -3’
	RP 5’- TCTCTTGCTCAGTGTCCTTGCT -3’

### Flow cytometry

CD8^+^ T-cells from differently treated mice groups were stained with PE-labeled TLR2, IFN-γ, IFN-γR, IL-12R, CD28 or IL-10, APC-Cy7 labelled CD25, FITC-lebelled IFN-γ. For intracellular cytokine staining, brefeldin A (10μg/mL) was added 4h prior to harvest, fixed, and permeabilized (0.1% saponin) and stained with anti-IFN-γ-PE, anti-perforin-PE and anti-granzyme-B-PE antibodies. Cells were analyzed using a FACS Verse flow cytometer.

### Isolation of RNA and Reverse Transcriptase polymerase chain reaction

Total RNA from purified CD8^+^ T-cells were extracted using TRI reagent using standard protocol [23]. The total RNA was reverse transcribed using Revert Aid M-MuLV reverse transcriptase (Fermentas). GAPDH was used as a loading control. Sequences of the PCR primers are given in the Table 1.

### CD8^+^ T-cell proliferation assay

Splenic CD8^+^ T-cells were cultured with autologus infected macrophages (10:1) for 72h and labellled with [^3^H]-thymidine (1μCi/10^5^ cells, JONAKI, DAE) for 18h before harvesting. [^3^H]-thymidine incorporation was determined using a liquid scintillation counter (Tri-Carb 2100TR; Packard Instrument) [24].

### Chromatin immunoprecipitation (ChIP) assay

ChIP assays were conducted using the ChIP Assay kit following the manufacturers protocol. Purified CD8^+^ T-cells (1×10^6^) from the indicated mice were co-cultured with autologous *L*. *donovani*–infected macrophages (1×10^5^) for 45 min, paraformaldehyde(1%)-fixed for 10 min at 37°C and washed with ice-cold PBS containing 1mM PMSF, harvested and lysed in SDS lysis buffer. DNA was sheared by ultrasonication using a High Intensity Ultrasonic Processor (Hielscher, Teltow, Germany) for 3 × 10s pulses at 20% amplitude. Lysates were cleared by centrifugation and diluted in ChIP dilution buffer. Lysates were pre-cleared using protein A-agarose and a sample of “input DNA” was collected at this point. Protein-DNA complexes were immunoprecipitated with 5μg of antibodies (phospho H3, acetyl H3, T-bet) overnight at 4°C. Antibody-protein-DNA complexes were then captured using protein A-agarose for 1 h at 4°C. After washing beads with low and high salt, LiCl, and TE buffers, the protein/DNA complexes were eluted in buffer (1%SDS, 0.1M NaHCO_3_). DNA was then extracted and precipitated. PCR was conducted using promoter specific primers (Table 1).

### Preparation of nuclear and cytoplasmic extracts

The nuclear extracts were prepared from CD8^+^ T-cells as described previously [25]. Briefly, cells were resuspended in ice-cold hypotonic buffer [10 mM HEPES (pH 7.9), 1.5 mM MgCl_2_, 10 mM KCl, 0.2 mM PMSF, and 0.5 mM DTT] for 10 min, homogenized and the nuclei were precipitated (3300×g, 5 min, 4°C). The supernatant was used as the cytoplasmic extract. The nuclear pellet was extracted in ice-cold nuclear extraction buffer [20mM HEPES (pH 7.9), 0.4M NaCl, 1.5mM MgCl_2_, 0.2mM EDTA, 25% glycerol, 0.5mM PMSF and 0.5mM DTT] for 30min (12,000×g for 30 min, 4°C). The supernatant was used as nuclear extract.

### Preparation of cell lysate and immunoblot analysis

CD8^+^ T-cells were lysed using lysis buffer for isolation of protein [26]. Equal amounts of protein (30μg) were subjected to 10% SDS-PAGE and transferred onto a nitrocellulose membrane. The membrane was blocked overnight with 3%BSA in Tris-saline buffer (pH 7.5), and immunoblotting was performed to detect MyD88, IRAK 1, NF-κB, T-bet, β-Actin, GAPDH, and phosphorylated or total p38MAPK and ERK1/2, as described previously [27].

### Coimmunoprecipitation

In coimmunoprecipitation studies, the lysates of differently treated CD8^+^ T-cells were incubated with specific antibody (TLR2, MyD88). The complexes were captured with immobilized Protein A-agarose beads, washed, resolved by 10% SDS-PAGE and developed with antibodies to MyD88 and IRAK 1 to detect TLR2-MyD88, MyD88-IRAK 1 interactions [28].

### Statistical analysis

All experiments were performed in triplicate and a minimum of 4 mice was used per group. The data, represented as mean values ± SD, are from one experiment that was performed at least 3 times. One-way ANOVA test (using a statistical package, Instat3) was employed to assess the significance of the differences. p≤0.001 was considered significant.

## Results

### Ara-LAM upregulates IL-12R and IFN-γR in CD8^+^ T-Cells in *L*. *donovani* infection

**Fig 1:**
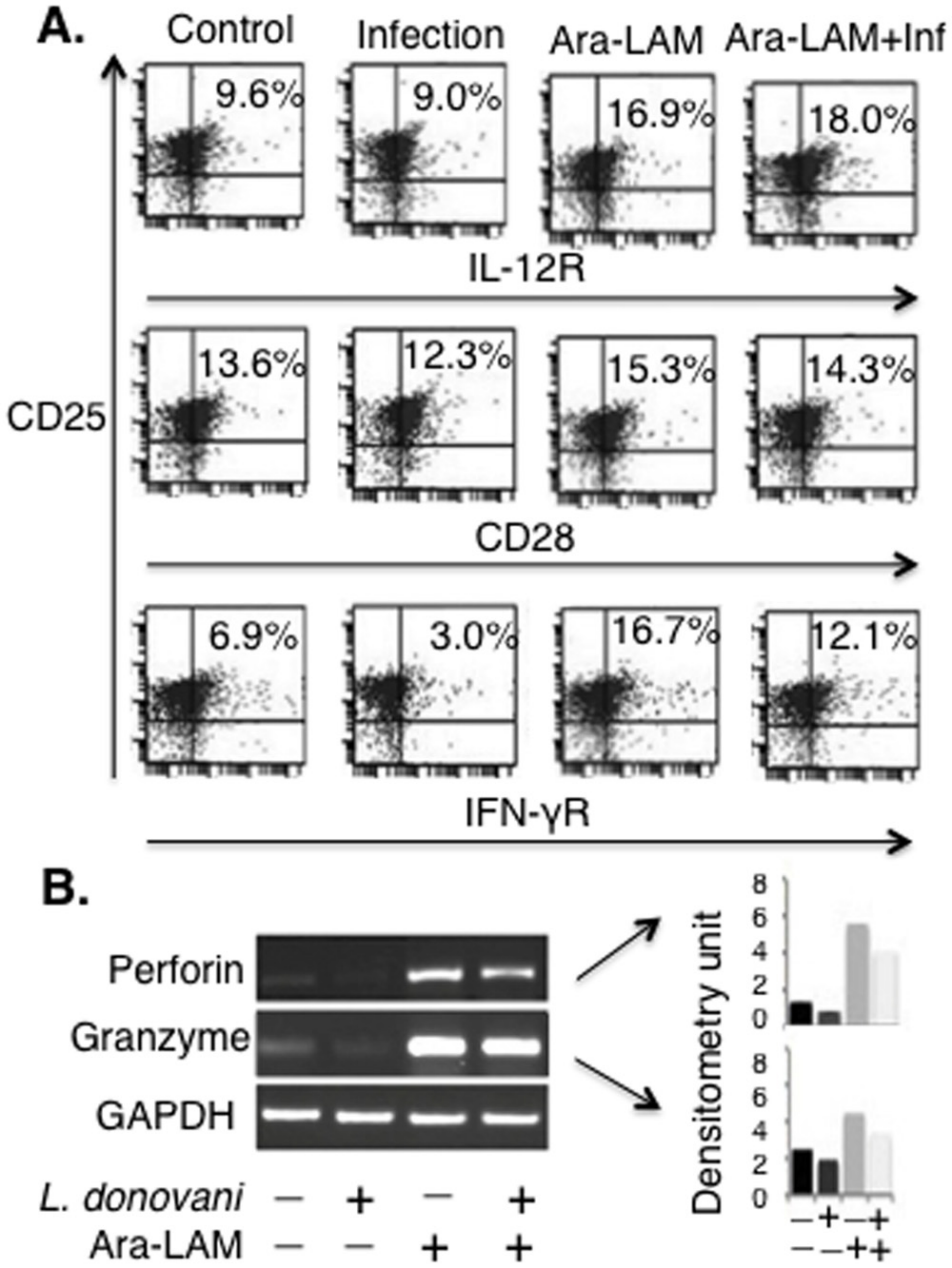
Characterization of CD8+ T cells at 28 days postinfection upon Ara-LAM treatment in *Leishmania donovani* infected BALB/c mice. (A) CD8^+^ T from differently treated BALB/c mice 28 days postinfection were subjected to FACS analyis to check the expression of CD25^+^IL12R^+^, CD25^+^CD28^+^, CD25^+^IFN-γR^+^ cells. Data are from one of three representative experiments. (B) In separate set of experiment, CD8^+^ T cells from differently treated mice group were isolated and cultured in presence of plate-bound anti-CD3ε mAbs (5μg/mL) and CD28 (1μg/mL) and expresion of perforin and granzyme-B was done by conventional RT PCR. Data are from one of three representative experiments.

We studied the effect of Ara-LAM on BALB/c mice-derived CD8^+^ T-cells in indicated groups. Naïve CD8^+^ T cells proliferate in response to TCR and CD28 signals, but reqiure IFN-γ and IL-12 to develop effector functions [29–30]. We investigated the status of CD28 on CD8^+^ T cells expressing CD25, receptor for IL-12 (IL-12R) and IFN-γ (IFN-γR) [31–32]. 28 days after infection, compared to the splenic CD8^+^ T cells of untreated infected mice, Ara-LAM strongly induced the expression of IL-12R and a moderate induction of IFN-γR on splenic CD8^+^ T cells, co-expresseing CD25 (Fig 1A). Activation of TLR2 in CD8^+^ T-cells is associated with their enhanced effecter functions [18–19]. Therefore, we tested whether Ara-LAM, being a TLR2 ligand, could activate the CD8^+^ T-cells by upregulating the transcription of perforin and granzyme-B. We observed a significant enhancement in both perforin and granzyme-B expression in CD8^+^ T-cells isolated from Ara-LAM treated *L*. *donovani* infected mice compared to that of untreated infected mice (Fig 1B).

### Ara-LAM-induced CD8^+^ T-cells activation in *L*. *donovani* infection is TLR2-dependent

**Fig 2:**
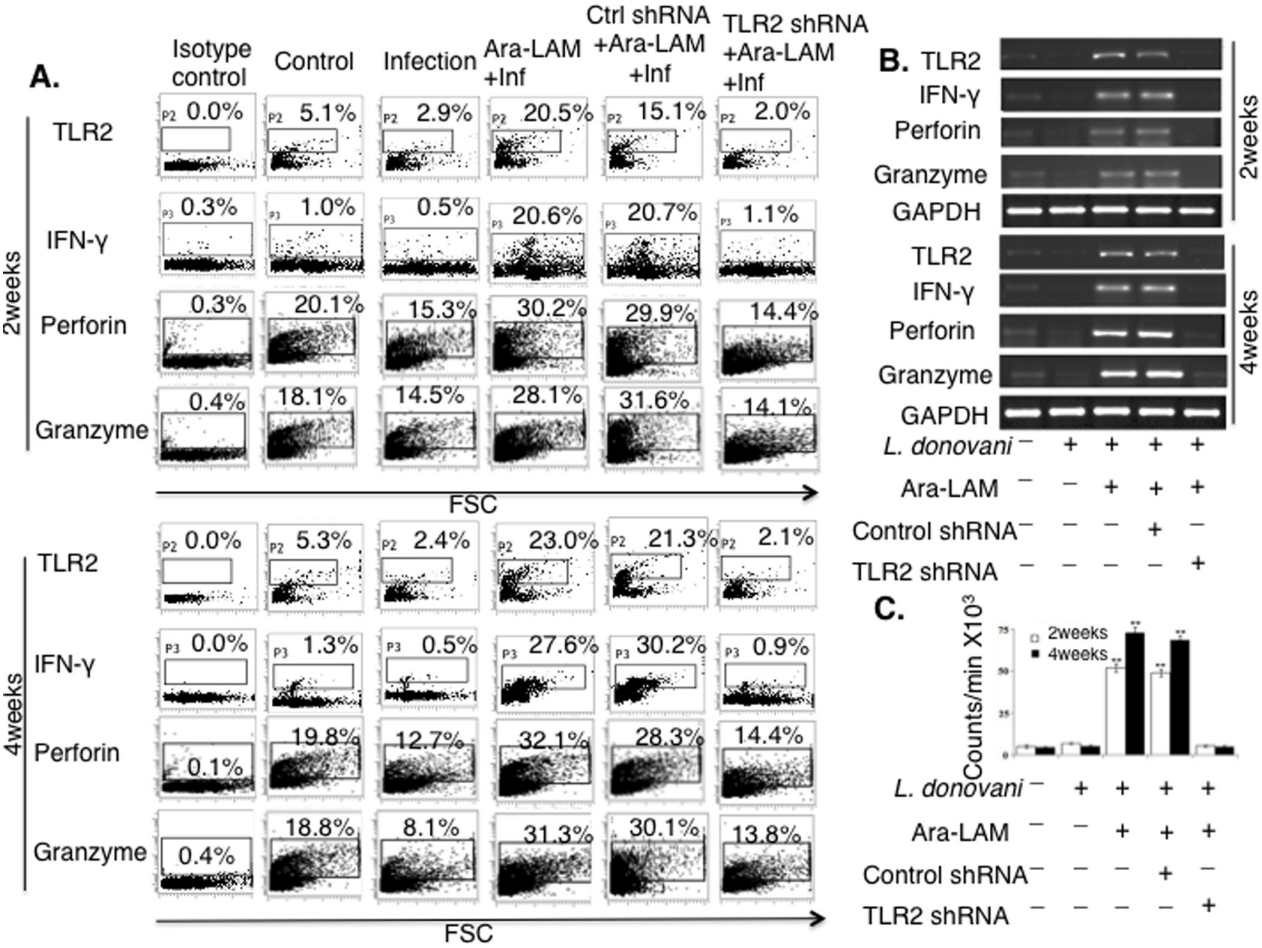
Ara-LAM facilitates TLR2 dependent activation and expansion of CD8^+^ T-cells in *Leishmania donovani* infected BALB/c mice. (A) Purified CD8^+^ T-cells were subjected to FACS analysis for TLR2 expression. Separately, purified CD8^+^ T-cells from differently treated mice were co-cultured with autologous infected macrophages (10:1) for 48hrs and IFN-γ, perforin, granzyme-B expression were determined by intracellular FACS. (B) CD8^+^ T-cells from differently treated mice groups were stimulated as described previously and conventional RT PCR was done after RNA extraction. (C) Purified CD8^+^ T-cells from differently treated mice and autologous *L*. *donovani*–infected macrophages were co-cultured for 72 hours. Proliferation was determined by an 18 h [^3^H] thymidine incorporation assay. Data were presented as count/million (×10^3^). Results were mean value ±SD. from triplicate wells. The asterisk indicated a statistically significant induction (**P<0.001) of T-cell proliferation, compared with that in infected mice.

We examined the effect of Ara-LAM treatment on TLR2 surface expression in CD8^+^ T-cells from different groups of BALB/c mice. Ara-LAM treatment significantly augmented the expression of TLR2 in splenic CD8^+^ T-cells on 14 and 28days post infection (Fig 2A). Because we observed significantly enhanced expressions of IFN-γ, perforin and granzyme-B in CD8^+^ T-cells isolated from Ara-LAM treated *L*. *donovani* infected mice compared to that of untreated infected mice (Fig 2A), we tested if TLR2 silencing could abrogate these effector functions. TLR2 silencing abrogated the Ara-LAM induced generation of IFN-γ, perforin, granzyme-B molecules in CD8^+^ T-cells isolated from the infected mice (Fig 2A and 2B).

It has been noted earlier that *Leishmania* infection of the susceptible host results in apoptosis of T-cells, leading to impairment of cell-mediated immunity [33]. Therefore, we investigated whether Ara-LAM could restore the impaired CD8^+^ T-cell proliferation in *Leishmania*-infected mice. Ara-LAM treatment resulted in significant enhancement of splenic CD8^+^ T-cell proliferation compared to the splenic CD8^+^ T-cells from untreated infected mice. The Ara–LAM–induced CD8^+^ T-cell proliferation was significantly attenuated during TLR2 silencing condition (Fig 2C). These results suggested that Ara-LAM stimulation leads to the enhanced effector function as well as the proliferation of CD8^+^ T-cells via TLR2 dependent pathway.

### Ara-LAM triggers T-bet recruitment and histone modification at the IFN-γ, Perforin and Granzyme-B promoter regions in CD8^+^ T-cells

**Fig 3:**
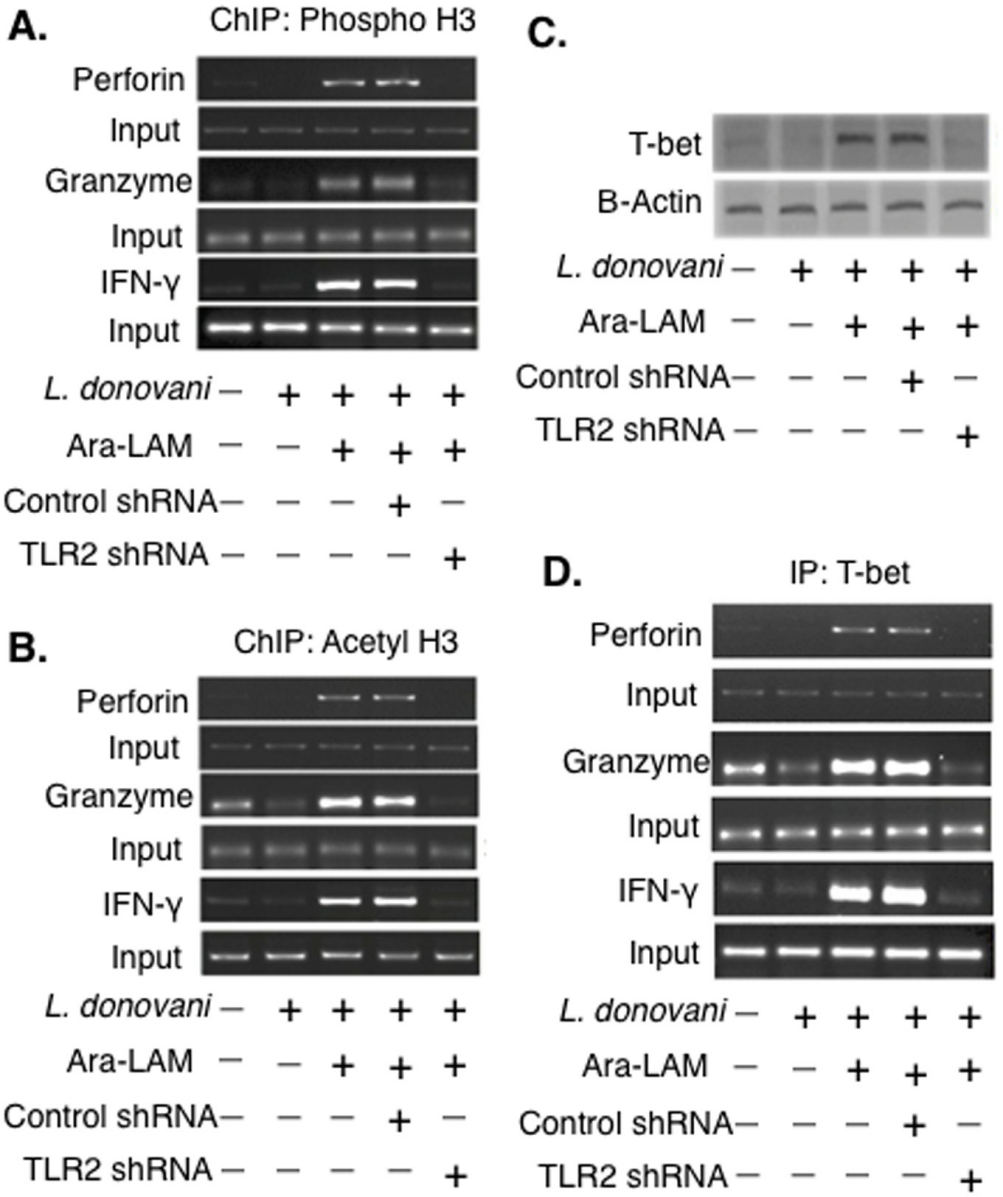
Histone H3 modifications at the IFN-γ, Perforin, Granzyme-B promoter of CD8^+^ T-cells in different groups of BALB/c mice. (A-B) CD8^+^ T cells from differently treated mice groups were co-cultured with autologous infected macrophages for 45 min and chromatin immunoprecipitation (ChIP) assays were conducted. Immunoprecipitations were performed using Abs specific to phosphorylated H3 histone (IP: phospho-H3), acetylated H3 histone (IP: acetyl-H3) and conventional RT PCR was performed using primers specific to the *IFN-γ*, *perforin* and *granzyme-B* promoter region. (C) CD8^+^ T-cells were co-cultured with infected macrophages, lysed and the nuclear protein extracts were analyzed for the activation of T-bet by Western blot. (D) CD8^+^ T-cells from differently treated mice group were co-cultured with autologous *L*. *donovani*–infected macrophages, Immunoprecipitations were conducted using T-bet (IP: T-bet) specific Abs. Conventional RT-PCR was performed for amplifying the putative T-bet binding sites of the *IFN-γ*, *perforin*, *granzyme-B* promoter. Data represented were one of the three indepenedent experiments with similar results performed in the same way.

The expressions of IFN-γ, perforin and granzyme-B in CD8^+^ T cells is principally regulated at the level of transcription, which, in turn, is strictly dependent upon the favourable histone modifications at their respective promoter regions [19]. Therefore, we performed ChIP assays to investigate whether the Ara-LAM-mediated enhancement of IFN-γ, perforin and granzyme-B expression in CD8^+^ T-cells was due to chromatin modification at their respective promoter regions. We observed a significantly higher level of phosphorylated (Fig 3A) and acetylated (Fig 3B) histones at the promoter regions of IFN-γ, perforin, granzyme-B in the splenic CD8^+^ T-cells of Ara-LAM treated *L*. *donovani* infected BALB/c mice relative to the splenic CD8^+^ T-cell from untreated infected mice. These Ara-LAM mediated histone modifications at the IFN-γ, perforin and granzyme-B promoter regions in CD8^+^ T-cells were significantly attenuated in TLR2 silenced condition (Fig 3A and 3B). Therefore, Ara-LAM induced transcription favourable histone modifications at the *IFN-γ*, *perforin*, and *granzyme-B* loci of CD8^+^ T-cells in a TLR2 dependent pathway.

The effector functions of CD8^+^ T-cells are predominantly regulated by the transcription factor T-bet [34]. Therefore, we investigated the accumulaion of T-bet in the splenic CD8^+^ T-cells of different experimental mice groups. Immunoblot analysis showed that accmulation of T-bet was significantly higher in the splenic CD8^+^ T-cells of Ara-LAM treated *L*. *donovani* infected mice compared to that of the untreated infected mice (Fig 3C).

T-bet binding at the promoter regions of IFN-γ, perforin, granzyme-B is a crucial event for the optimal induction of these effectors molecules in CD8^+^ T-cells [13]. Therefore, we performed ChIP-on-ChIP assay to investigate the interaction of T-bet with the promoter regions of IFN-γ, perforin, and granzyme-B in CD8^+^ T-cells from Ara-LAM treated and untreated *L*. *donovani* infected BALB/c mice. Ara-LAM treatment induced a strong association of T-bet with the IFN-γ, perforin, and granzyme-B promoter regions in CD8^+^ T-cells compared with that of the untreated *L*. *donovani* infected mice; TLR2 silencing decreased Ara-LAM induced T-bet recruitment to the promoter regions of IFN-γ, perforin, granzyme-B in CD8^+^ T-cells (Fig 3D). Collectively, Ara-LAM-induced TLR2 dependent activation of T-bet augmented the transcription of target effector genes in CD8^+^ T-cells of infected mice.

### Ara-LAM promotes NF-κB activation and MAPK signaling in CD8^+^ T-cells

**Fig 4:**
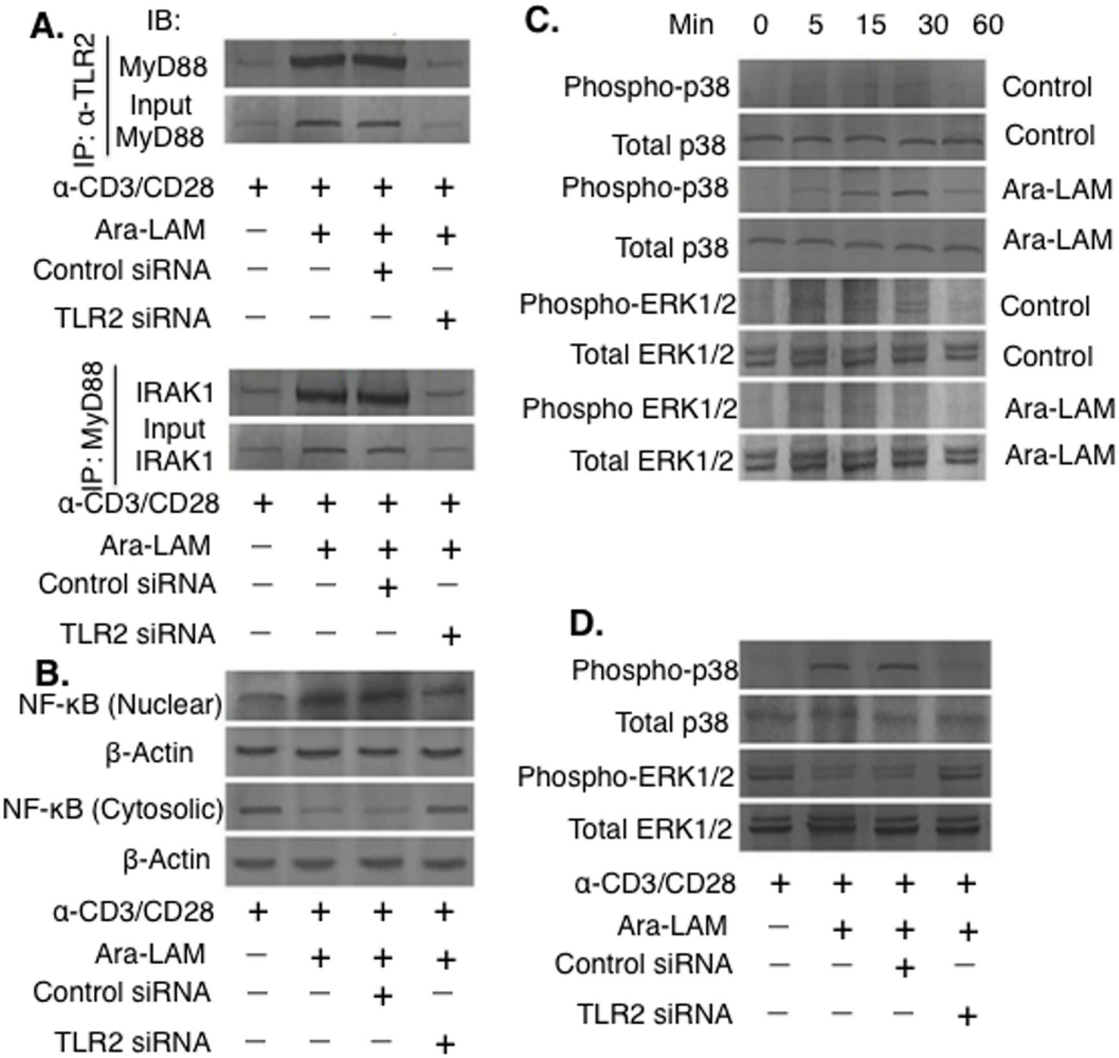
Ara-LAM activates TLR2 signalling via activation of NF-κB and p38 MAPK in naive CD8^+^ T-cells. (A) Purified CD8^+^ T-cells (1×10^6^/mL) were stimulated with plate-bound anti-CD3ε mAbs (5μg/mL) and CD28 (1μg/mL) for 24 hrs and transfected with control siRNA or TLR2 siRNA, followed by Ara-LAM (3μg/mL) treatment for 24 hr. The cells were then lysed and subjected to immunoprecipitation with anti-TLR2 antibody, and the blots were probed with anti-MyD88 antibody. (B) Cells were lysed and immunoprecipitated with anti-MyD88 antibody; the blots were probed with anti-IRAK1 antibody. Cytosolic and nuclear protein extracts were analyzed for nuclear translocation of NF-κB. (C-D) In yet separate experiments, CD8^+^ T cells were treated by Ara-LAM for 5, 15, 30, and 60 min, and lysed. The lysate was subjected to Western blot analysis for the expression of p38MAPK, phospho-p38MAPK and ERK1/2, phospho-ERK1/2. Data represented were one of the three indepenedent experiments with similar results performed in the same way.

Ara-LAM triggers the TLR2 mediated downstream signaling in host macrophages [18–19]. However, Ara-LAM induced modulation of TLR2 signaling in CD8^+^ T-cells has not been explored till date. The association between TLR2 and MyD88 is a crucial event for the initiation of TLR2 downstream signaling [18]. Therefore, we carried out co-immunoprecipitation studies to investigate the TLR2-MyD88 interaction in Ara-LAM–treated CD8^+^ T-cell. We observed a strong association between TLR2 and MyD88 in Ara-LAM–treated CD8^+^ T-cell compared with that of the untreated CD8^+^ T-cells. IRAK-1, crucial for activation of TLR2 downstream signaling [18], was found to be intricately associated with MyD88 in Ara-LAM–treated CD8^+^ T-cells compared to the untreated CD8^+^ T-cells (Fig 4A). Furthermore, nuclear translocation of NF-κB were significantly augmented in Ara-LAM treated CD8^+^ T-cell compared to the untreated CD8^+^ T-cells. Ara-LAM induced TLR2 downstream signaling mediated NF-κB translocation was completely abrogated under TLR2 silenced condition (Fig 4B).

In addition to the NF-κB activation, TLR2 signaling can also modulate p38 and ERK-1/2 MAPK signaling cascades [19]. Therefore, we investigated whether Ara-LAM treatment could induce MAPK phosphorylation in CD8^+^ T-cells. Ara-LAM treatment resulted in significantly higher level of p38MAPK phosphorylation (Fig 4C), however; it failed to induce significant ERK1/2 phosphorylation in CD8^+^ T-cells (Fig 4C). TLR2 silencing abrogated the Ara-LAM induced p38 phosphorylation (Fig 4D). Taken together, Ara-LAM activated p38MAPK and NF-κB activation in infected CD8^+^ T-cells.

### Ara-LAM enhances T-bet expression in CD8^+^ T-cells via upregulation of NF-κB and p38MAPK signaling

**Fig 5:**
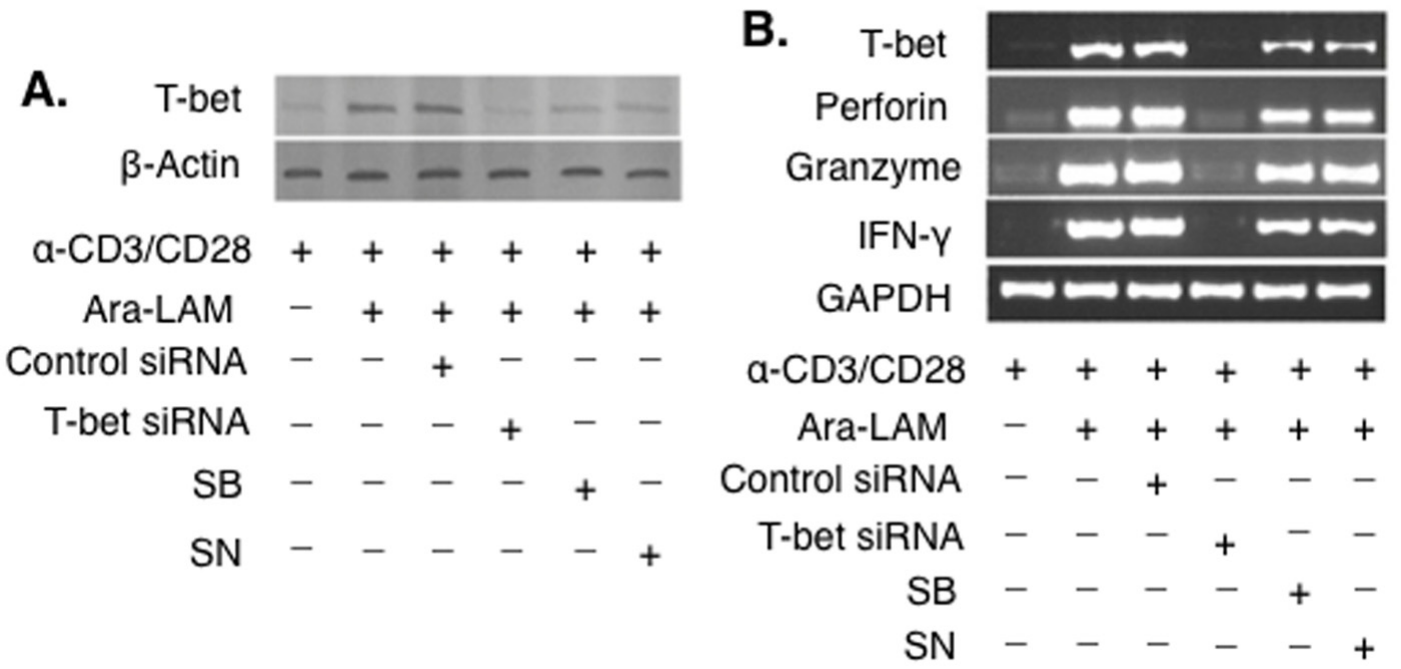
Ara-LAM promptly regulates effector functions in CD8^+^ T-cells through NF-κB and p38MAPK mediated T-bet signalling. (A) CD8^+^ T-cells were isolated by MACS from the spleen BALB/c mice. Purified CD8^+^ T cells were stimulated as described previously and allowed to transfect with control siRNA or T-bet siRNA, or treated with SB203580 (SB) (5μg/ml), or SN50 (SN) (20μg/ml), subsequently followed by Ara-LAM (3μg/mL) treatment for 24 hr. The cells were then lysed and nuclear protein extracts were prepared, followed by subjected to Western blot with anti-T-bet. (B) The blot shown is representative of triplicate experiments that yield similar type of results. In a separate set of experiments, after the treatment schedule, the cells were collected in Trizol for RNA extraction, and conventional RT PCR analysis was performed to determine the expression of T-bet, IFN-γ, perforin, granzyme-B. Data represented were one of the three indepenedent experiments with similar results performed in the same way.

Since, Ara-LAM induced efficient activation of T-bet in CD8^+^ T-cells (Fig 3C) and enhanced the activation of NF-κB and p38MAPK in CD8^+^ T-cell (Fig 4), we hypothesized that T-bet activation in CD8^+^ T-cells might be regulated either by the NF-κB or MAPK signalling or by both of these signalling molecules. Ara-LAM mediated activation as well as mRNA expression of T-bet was attenuated in the presence of SB203580 and SN50, the pharmacological inhibitors of p38MAPK and NF-κB, respectively (Fig 5A and 5B), suggesting that the activation of T-bet in Ara-LAM treated CD8^+^ T-cells was regulated by TLR2 triggered NF-κB and p38MAPK signalling cascade. Besides, the enhanced IFN-γ, perforin and granzyme-B expression in Ara-LAM stimulated CD8^+^ T-cells were significantly decreased during T-bet silencing, as well as, NF-κB and the p38MAPK inhibition (Fig 5B). Therefore, the upregulation of IFN-γ, perforin and granzyme-B expression in Ara-LAM stimulated CD8^+^ T-cells were due to the NF-κB and the p38MAPK dependent activation of T-bet.

### Ara-LAM reduces hepatic and splenic parasitic burden in BALB/c mice

**Fig 6:**
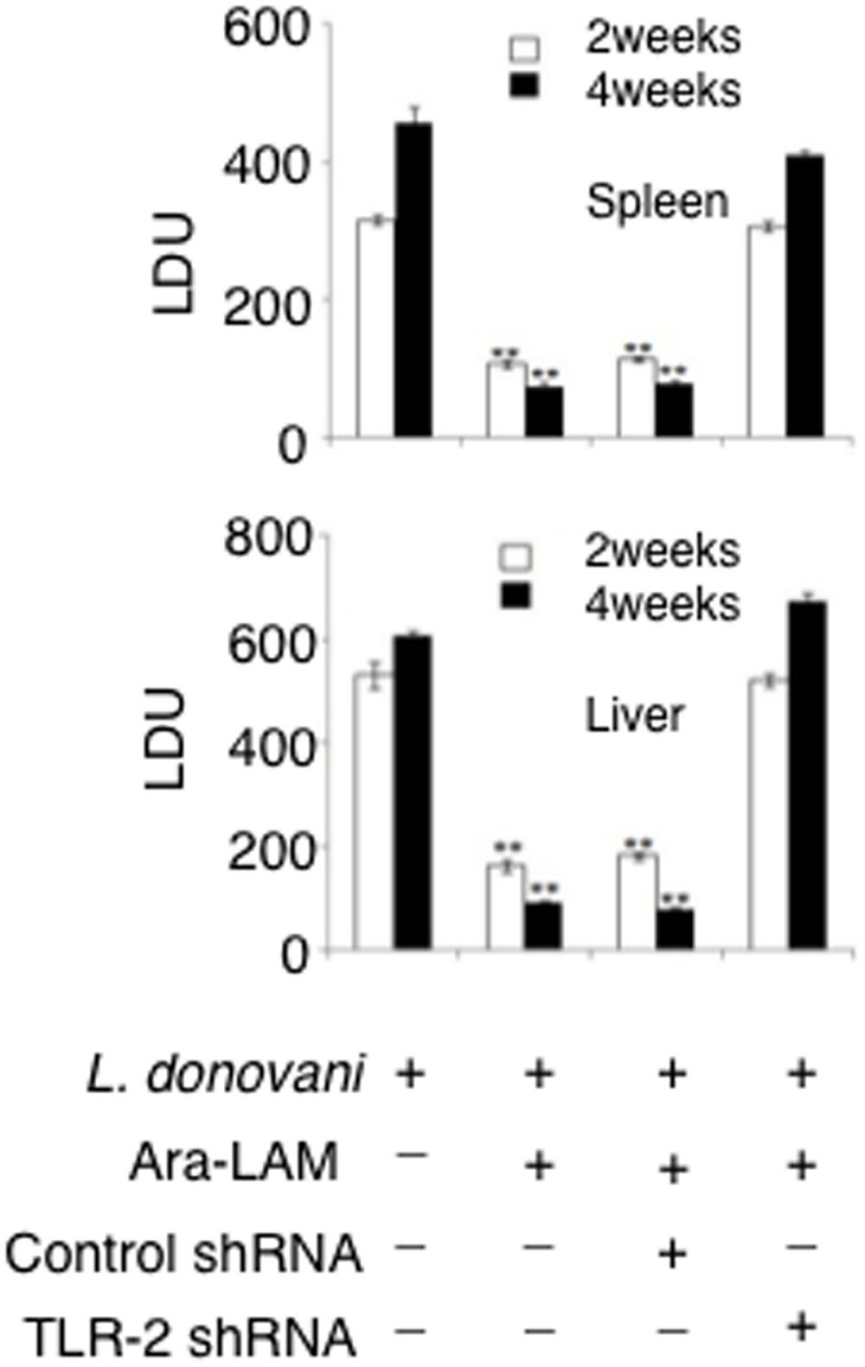
Ara-LAM treatment in *L*. *donovani* infected BALB/c mice showed reduced parasite burden. Mice were treated as described in Fig 1 legend. Mice from different groups were sacrificed on day 14 and 28 post-infection. The parasite burden in liver and spleen were expressed in Leishman-Donovan units (LDUs). Results were for 3 independent experiments and represented the mean values ± SD for four animals per group. **P<0.001 for the comparison with infected mice.

We have observed that Ara-LAM activates the CD8^+^ T cells via upregulation of IFN-γ and the cytotoxic molecules perforin and granzyme-B. These activated T cells have the ability to kill the infected macrophages to reduce the parasite burden [5–6]. In accordance with this phenomenon, we observed a significant reduction of the hepatic as well as splenic parasite burden in the Ara-LAM treated infected mice groups compared to the untreated infected mice groups (Fig 6) on 14 and 28days postinfection. However, Ara-LAM mediated clearance of parasites was significantly attenuated in TLR2 silenced condition. Collectively, the data clearly suggest that Ara-LAM plays a very important role to clear the *L*. *donovani* infection via a TLR2 dependent mechanism.

### Reversion of the Th subset expansion in *L*. *donovani* infected mice

**Fig 7:**
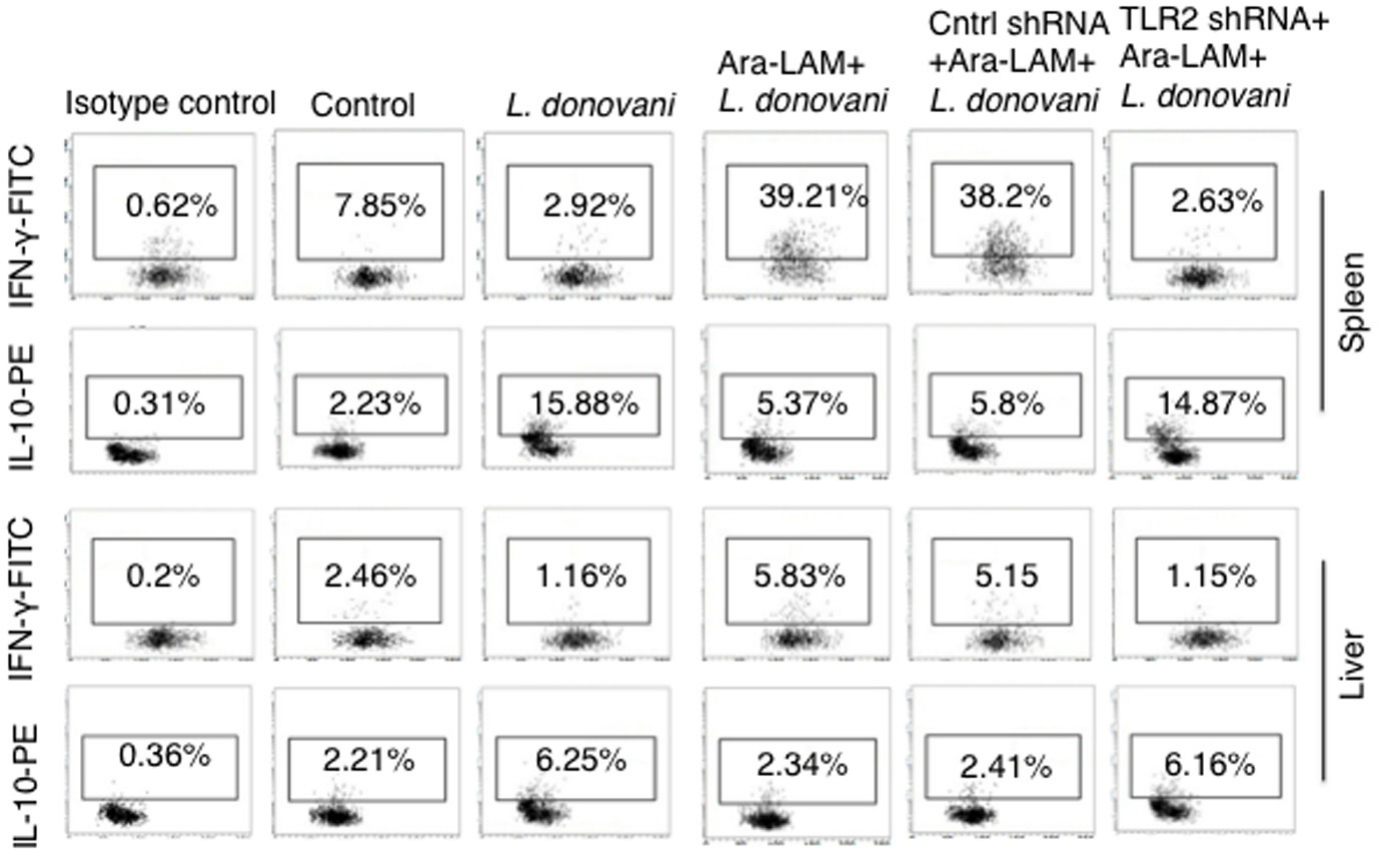
Ara-LAM up-regulates IFN-γ secreting CD8^+^ T-cells in an organ-dependent manner during *L*. *donovani* infection. 28days post-infection, different groups of mice were sacrificed. Splenocytes and hepatocytes were stimulated with soluble leishmanial antigen (SLA, 10 μg/mL) for 48hrs. Before harvesting, cells were incubated with brefeldin A (10 μg/mL) for 4 hrs, CD8^+^ T cells were MACS sorted (see Materials and Methods), permeabilized (0.1% saponin) and stained with anti-mouse IFN-γ-FITC and anti-mouse IL-10-PE antibodies and were analyzed by flow cytometry. Data are from 1 of 3 experiments conducted in the same way with similar results.

We checked the effect of Ara-LAM on IFN-γ and IL-10 producing-CD8^+^ T-cells in both spleen and liver by flow cytometry. In both uninfected and infected mice, Ara-LAM up-regulated the IFN-γ secreting CD8^+^ T-cells in spleen as well as liver. IL-10 producing CD8^+^ T-cells were suppressed by Ara-LAM pretreatment in both spleen and liver of infected as well as uninfected mice. Although in case of hepatic CD8^+^ T-cells, the effect of Ara-LAM on both IFN-γ and IL-10 secretion was not very significant (Fig 7).

## Discussion

Leishmanial pathogenesis is associated with abrogated pro-inflammatory responses, resulting in immune suppression of the host [9–11]. The persistence of the infection during VL was due to the impaired cell-mediated immunity which in turn was intricately associated with the severe dysfunction of cytotoxic CD8^+^ T-cells [7]. Therefore, activation of CD8^+^ T cells is very much important to eradicate *L*. *donovani* mediated infection. Recently, it has been reported that activation of TLR signalling could restore the impaired effector function of CD8^+^ T-cells in various models of infectious diseases [35]. Therefore, we intended to study whether Ara-LAM, so far known to be a TLR2 ligand [18], could restore the functional capacity of the effector CD8^+^ T-cells during VL.

We confirmed that Ara-LAM pre-treatment in *L*. *donovani* infected BALB/c mice significantly augmented the expression of TLR2 along with the concomitant increase in IFN-γ, perforin, and granzyme-B in the splenic CD8^+^ T-cells (Figs 1 and 2). Because knowing phenotype is important for its functional significance, we examined the phenotype of these T cells, which are IL-12R^+^IFN-γ^+^CD28^+^ co-expressing CD25 (Fig 1A). Moreover, Ara-LAM pre-treatment was found to be associated with significant enhancement of CD8^+^ T-cell proliferation (Fig 2C), a prerequisite for its effector function. TLR2 silencing significantly attenuated such Ara-LAM mediated enhanced expression of these effector molecules in CD8^+^ T-cells (Fig 2).

The optimal transcriptional induction of the IFN-γ, perforin, granzyme-B genes in CD8^+^ T-cells requires histone modification at their respective promoter regions [19]. Our study revealed that Ara-LAM pretreatment led to transcription favourable histone H3 phosphorylation and acetylation specifically at the promoter region of IFN-γ, perforin, granzyme-B in the splenic CD8^+^ T-cells of *L*. *donovani*-infected BALB/c mice (Fig 3A and 3B). In line with the fact that the activation of the transcription factor T-bet is a crucial event for the expression of IFN-γ, perforin, granzyme-B in CD8^+^ T-cells [13, 34], we observed an increased T-bet accumulaion (Fig 3C) along with enhanced T-bet binding to the promoter regions of IFN-γ, perforin, granzyme-B in the Ara-LAM treated splenic CD8^+^ T-cells of *L*. *donovani* infected BALB/c mice (Fig 3D). Hence, our study revealed that Ara-LAM induces T-bet dependent activation of effector molecules in CD8^+^ T-cells to hinder leishmanial pathogenesis.

Further, we intended to investigate the underlying molecular mechanism of Ara-LAM induced CD8^+^ T-cells activation during VL. Since, Ara-LAM confers its protective functions against VL via activation of the TLR2 downstream signaling in host macrophages [18–19], we investigated Ara-LAM mediated modulation of TLR2 signaling in CD8^+^ T-cells. Ara-LAM treatment led to successful initiation of TLR2 signalling in naive CD8^+^ T-cells via the TLR2-MyD88 association (Fig 4A), resulting in the selective activation of intermediate signalling molecules, IRAK1 and ultimately leading to nuclear translocation of NF-κB (Fig 4B). Ara-LAM treatment also led to increased p38MAPK phosphorylation along with concomitant attenuation of ERK1/2 phosphorylation in splenic CD8^+^ T-cells (Fig 4C and 4D). These observations indicate that Ara-LAM induced activation of TLR2 downstream signalling molecules leads to enhanced effector function of CD8^+^ T-cells during VL.

We observed that inhibition of NF-κB and p38MAPK, by their respective pharmacological inhibitors, significantly abrogated the Ara-LAM–induced T-bet expression and activation in CD8^+^ T-cells (Fig 5A and 5B). Our results indicated the active involvement of NF-κB and p38MAPK signalling in the regulation of Ara-LAM driven T-bet activation. Further, Ara-LAM treatment significantly reduced the parasite burden in the liver and spleen 14 and 28 days after the infection (Fig 6) accompanied with the expansion of IFN-γ^+^CD8^+^T-cells (Fig 7). It is therefore feasible to conclude that Ara-LAM mediated reduced parasitic burden may be due to the CD8^+^ T-cell driven killing of the infected macrophages. It is noteworthy to mention that Ara-LAM works well when utilized as an immunotherapiutic agent administered 2days prior to *L*. *donovani* infection [18–20, 28].

In summary, Ara-LAM confers significant protection through activation of CD8^+^ T-cells in *L*. *donovani* infected BALB/c mice. The novelty of our work lies in the fact that Ara-LAM-induced TLR2 signaling leads to the activation of the transcription factor T-bet which plays a pivotal role in restoring the effector functions of CD8^+^ T-cells in *L*. *donovani* infected susceptible host. Thus, we are one the way to devise a strategy in near future so that Ara-LAM can be used as a suitable vaccine during *L*. *donovani* infection.
