# Expression of Concern: Toll-Like Receptor 2 Targeted Rectification of Impaired CD8^+^ T Cell Functions in Experimental *Leishmania donovani* Infection Reinstates Host Protection

**DOI:** 10.1371/journal.pone.0347711

**Published:** 2026-04-22

**Authors:** The PLOS One Editors

After this article [1] was published, concerns were raised regarding results presented in Figs 4 and 7.

Specifically:

- The following panels in Fig 4C appear similar:
  - Phospho-ERK1/2 Control and Phospho ERK1/2 Ara-LAM
  - Total ERK1/2 Control and Total ERK1/2 Ara-LAM
- The Fig 7 Liver IFNγ-FITC *L. donovani* panel and the Fig 7 Liver IFNγ-FITC TLR2 shRNA + Ara-LAM + *L. donovani* panel appear more similar than would be expected from independent results.
- Contrary to the article’s Data Availability statement, the original raw data files supporting the article’s results were not provided with the article. As the original raw data files supporting the article’s results have not been made available, this article [1] does not comply with the PLOS Data Availability policy in place at the time this article was submitted.

In addition, following publication of this article, concerns were raised about a potential competing interest between the authors and the Academic Editor who handled the peer review for this article. PLOS regrets that this issue was not addressed prior to the article’s publication.

The authors did not respond to editorial request for comment.

The *PLOS One* Editors issue this Expression of Concern to inform readers of the above unresolved concerns and to inform readers that the results presented in Figs 4 and 7 should be interpreted with caution.
